# Isolation of antibodies specific to a single conformation-dependant antigenic determinant on the EG95 hydatid vaccine

**DOI:** 10.1016/j.vaccine.2008.11.096

**Published:** 2009-02-11

**Authors:** A.J. Read, J.L. Casey, A.M. Coley, M. Foley, C.G. Gauci, D.C. Jackson, M.W. Lightowlers

**Affiliations:** a The University of Melbourne, Veterinary Clinical Centre, 250 Princes Highway, Werribee, Victoria 3030, Australia; b The University of Melbourne, Department of Microbiology and Immunology, Parkville, Victoria 3010, Australia; c AdAlta Pty Ltd., Department of Biochemistry, La Trobe University, Victoria 3086, Australia

**Keywords:** *Echinococcus granulosus*, Hydatid, Random peptide phage display, Conformation-dependant epitope

## Abstract

EG95 is a recombinant vaccine protein that elicits protection against hydatid disease in sheep. Previous studies have shown that the host-protective epitopes on EG95 depend on correct conformation and cannot be represented by simple “linear” peptides. By screening random peptide phage display libraries with polyclonal antibodies directed against conformation-dependant epitopes of EG95, we have selected a number of peptides that mimic these epitopes. The selected peptides did not show sequence homology to EG95. Antigen binding assays involving these peptides have provided evidence of at least four conformationally-dependant epitope regions on EG95. One of the selected peptides, E100, has been used to purify antibodies from anti-sera raised in sheep vaccinated with EG95. This yielded monospecific antibodies capable of recognizing recombinant EG95 in ELISA and native EG95 in Western blot assays. This antibody was demonstrated to be effective in antibody-dependant complement-mediated *in vitro* killing of *Echinococcus granulosus* oncospheres. Peptide E100 may represent the basis for a quality control assay for EG95 production, and has the potential to become a component of a synthetic peptide-based vaccine against *E. granulosus*.

## 1 Introduction

The metacestode life stage of the taeniid cestode *Echinococcus granulosus* is the aetiological agent of cystic hydatid disease (CHD) of humans. CHD is a disease which often affects rural communities, with a global distribution [1]. Surveys involving populations where the disease is highly endemic have shown prevalence in the order of 2% to 6%. Although mortality directly associated with CHD is not high, there is often significant morbidity. A number of researchers have documented the impact of the disease in terms of human health and financial losses to communities [2]. The dog is the definitive host in the life cycle of the parasite, while various herbivorous mammals, especially sheep, become infected as intermediate hosts. Humans are infected by fortuitously ingesting parasite eggs present in the faeces of the definitive host.

A recombinant vaccine has been developed for prevention of infection in the intermediate host [3] and the bacterially expressed recombinant antigen is designated EG95. EG95 has been shown to induce between 95% and 100% protection in sheep against a challenge infection with *E. granulosus* eggs [3], and has proved to be highly effective in preventing infection in New Zealand, Australia and Argentina [4]. The EG95 vaccine developed for sheep may be a useful tool to reduce the incidence of transmission of hydatid disease to humans in endemic areas [5].

There is significant evidence that antibody-mediated complement-dependant lysis of oncospheres is the major mode of protection against infection amongst the taeniid cestodes [6]. Immunity can be passively transferred from a previously infected animal or animal vaccinated with early larval (oncosphere) antigens via colostrum or whole serum [5]. Sheep immunised with either *E. granulosus* oncospheres [7] or the EG95 antigen [3,8] produce antibodies which are capable of killing *E. granulosus* oncospheres *in vitro*. Taken together these pieces of evidence suggest that anti-EG95 antibodies play a crucial role in vaccine-induced protection.

It has been predicted from the amino acid sequence of EG95 that the protein is dominated by a fibronectin type III (FnIII) domain [9]. Although linear epitopes on the protein have been identified [10] immunisation with peptides representing these linear epitopes [11] or larger polypeptides lacking the complete FnIII domain [8] failed to induce protection against infection. It has been postulated that the protective epitope(s) of EG95 are dependant on the correct conformation of EG95 and cannot therefore be properly represented by simple peptide sequences, furthermore the important epitopes most likely involve the FnIII domain [8].

By inserting a randomised nucleotide sequence of a given length into M13 filamentous bacteriophage (phage) DNA it is possible to produce random peptide phage display (RPPD) libraries which can display many millions of different peptides. Such RPPD libraries have been used to select peptides that bind specifically to a given target [12] enabling the identification of “mimotopes” that mimic a conformation-dependant epitope [13]. Mimotopes often lack any sequence homology to the original antigen, but nevertheless mimic the three dimensional structure of the epitope thereby allowing recognition and binding of antibody elicited by the original protein antigen. Phage displayed peptides have been usefully applied in a number of vaccine-related studies [14] including studies involving a cestode parasite [15].

The identification of ligands that are capable of mimicking the protective epitope(s) of EG95 may assist in quality control measures for the production of the recombinant vaccine and could even lead to the development of a totally synthetic vaccine against CHD. Previous attempts to identify mimotopes of conformation-dependant epitopes of EG95 (11) have been unsuccessful resulting in the selection of peptides that correspond to linear sequences of amino acids within EG95 and which represent non-protective epitopes. In this study we have applied the strategy of screening two RPPD libraries with polyclonal antibodies that are specifically directed against the conformation-dependant epitope(s) of the protein. We show that antibodies directed against conformational-dependant epitope(s) may be purified from the serum of sheep immunised with EG95 and that by screening these antibodies against RPPD libraries, peptides may be selected that allow the purification of antibodies against a single conformation-dependant epitope.

## 2 Materials and methods

### 2.1 Source of antibodies

The isolation and characterisation of polyclonal antibodies against conformational epitopes of EG95 have been described elsewhere (A.J. Read, C.G. Gauci and M.W. Lightowlers, under review). Briefly, pooled anti-sera were derived from sheep immunised with EG95-GST. Affinity chromatography was used to deplete the sera of antibody specificities to GST and linear epitopes of EG95. The sera depleted of these specificities were then affinity purified on an EG95-GST chromatography column. Antibodies eluted from this column were used in affinity selection experiments using RPPD libraries and referred to as anti-cEG95 antibodies. The GST affinity purification column used in the depletion step was used to generate polyclonal anti-GST antibodies (anti-GST).

### 2.2 Source of random peptide phage display libraries

Two non-constrained random peptide phage display libraries were used to select peptides bound by anti-cEG95. These are the Ph.D-12 Phage Display Kit (New England Biolabs, USA) [16] (Library 1) and a 20-mer library (Library 2, provided by AdAlta Pty Ltd) [17]. Each library carried a random DNA insert encoding a peptide near the N terminus of the pIII minor coat protein at one end of the filamentous phage. The Ph.D-12 library is based on the M13KE phage vector and expresses a 12-mer peptide while the 20-mer library is based on the fUSE5 vector [12] and expresses a 20-mer peptide.

### 2.3 Affinity selection

The affinity selection of phage that is specifically bound by anti-cEG95 was conducted in a manner similar to that described elsewhere [17]. Anti-cEG95 antibodies or anti-GST antibodies in 100 μl were adsorbed overnight at 4 °C onto ten wells in a microtitre plate (Maxisorp, NUNC, Denmark). The wells were then emptied and blocked for 2 h with 5% skim milk powder in phosphate buffered saline (145 mM NaCl, 2.7 mM KCl, 12 mM Na_2_HPO_4_, 1.2 mM KH_2_PO_4_, pH 7.4) (SMPBS). Approximately 11 × 10^10^ transforming units (TU) of phage per ml was pre-incubated for 1 h in SMPBS and following removal of the blocking agent, 100 μl of the phage solution was added to each well for a period of 2 h. The solution containing phage was then removed and the wells were washed. The stringency of selection was increased with each round of affinity selection by increasing the number of washes performed. Bound phages were eluted from the wells by the addition of 100 μl 0.1 M Glycine/HCl, pH 2.2 for 10 min. The eluate was pooled and neutralised with 1 M Tris. Eluted phages were amplified using *E. coli* (ER2537) for Library 1 and *E. coli* (K91) for Library 2. A total of four rounds of affinity selection were undertaken for each library. The DNA sequence of isolated phage clones was determined using an Applied Biosystems PRISM™ automated sequencing system with the ABI BigDye™ Terminator sequencing kit. The amino acid sequence of each clone was deduced from the DNA sequence.

### 2.4 Phage indirect ELISA

Affinity purified antibodies (anti-cEG95 and anti-GST) were adsorbed onto microtitre plates overnight at 4 °C. Non-specific binding was blocked by adding SMPBS for 1 h at room temperature. Phages were added to the washed plates either at a set concentration or following serial dilution in SMPBS. Phages were left for 1 h at room temperature. Levels of phage binding were assessed through the addition of a mouse monoclonal anti-M13 HRP (Pharmacia, Sweden). Peroxidase activity was detected by adding a tetramethyl benzidine (TMB) substrate (Sigma, USA) to the test wells for 30 min before stopping the reaction with 0.5 M H_2_SO_4_. Absorbance was measured at 450 nm.

### 2.5 Antibody indirect ELISA

Recombinant proteins EG95-MBP (EG95 bound to Maltose Binding Protein affinity tag) or GST were adsorbed onto microtitre plates overnight at 4 °C. Non-specific binding was blocked by adding SMPBS for 1 h at room temperature. Serially diluted serum or affinity purified antibody samples were added to the wells for 1 h at room temperature and levels of IgG were assessed by the addition of a polyclonal donkey anti-sheep IgG-HRP (Sigma, USA) to each well for 1 h at room temperature. Peroxidase activity was detected by adding a TMB substrate to the test wells for 30 min before stopping the reaction with 0.5 M H_2_SO_4_. Absorbance was measured at 450 nm.

### 2.6 Synthesis of peptides

Solid-phase peptide syntheses were performed using 9-fluorenylmethoxycarbonyl (Fmoc) chemistry either manually or in an automated solid-phase synthesizer (Symphony™ automated peptide synthesizer, Rainin-PTI Woburn, USA) on a cleavable resin (TentaGel S-RAM, Rapp Polymere GmbH, Germany). Peptides were analysed by reverse phase chromatography using Vydac C8 columns (4.6 mm × 250 mm) installed in a Waters HPLC system and subsequently purified by reverse phase chromatography using Vydac C18 column (10 mm × 300 mm).

The peptide E3, yvalqgsmfdrvrvfwmarg, was found to be insoluble in aqueous solution. Solubility was improved by adding five lysine residues to the amino terminus. This peptide is referred to as K_5_-E3.

### 2.7 Affinity purification using E100/G1

Activated NHS-Sepharose^®^ was prepared according to the manufacturer’s instructions. Briefly, pre-swollen NHS-Sepharose beads were mixed with free synthetic peptides E100 and G1 dissolved in coupling buffer (0.2 M NaHCO_3_, 0.5 M NaCl, pH 8.3). Remaining activated sites on the beads were blocked with Tris buffered saline (145 mM NaCl, 20 mM Tris, pH 7.4).

Pooled serum from sheep immunised with EG95-GST was diluted in an equal volume of binding buffer (0.01 M Na_2_HPO_4_, 0.15 M NaCl, 0.01 M EDTA, pH 7.0). The anti-serum solution was applied to the affinity columns under gravity. Unbound material was removed by washing columns with up to 50 ml of binding buffer. Bound antibodies were eluted under low pH conditions (0.1 M Glycine/HCl, 0.15 M NaCl, pH 2.6). The eluted fractions were collected and adjusted to pH 7.5. The fractions with indirect antibody ELISA absorption values >0.75 were pooled.

### 2.8 Immuno-blots of recombinant proteins

Nitrocellulose 0.2 μm (Hybond™ ECL™, GE Healthcare, USA) was pre-wetted in Towbin Transfer Buffer (25 mM Tris, 193 mM glycine, 20% methanol, pH 8.4). Protein concentrations were determined using the Biorad Protein Assay (Bio-Rad, USA). One microgram of each of the recombinant proteins EG95-GST, GST and EG95-MBP was pipetted on to the nitrocellulose. The blot was dried and incubated for 1 h in SMPBS-0.05% Tween (SMPBST) to block non-specific binding sites. Incubation with primary antibodies was carried out for 1 h at room temperature. Whole serum pools taken from sheep prior to and after immunisation with EG95-GST were diluted 1:2000 in SMPBST. Affinity purified antibodies were diluted to 1 μg/ml in SMPBS. The membranes were subsequently incubated for 1 h at room temperature with a 1:2000 dilution of a polyclonal donkey anti-sheep IgG-HRP conjugate diluted in SMPBST. The antibodies on the paper were detected and visualised by Enhanced Chemiluminescence (ECL; Boehringer Mannheim, Germany).

### 2.9 Western blots of native antigens

*E. granulosus* oncospheres were harvested, hatched and activated [7]. The oncospheres were solubilised in SDS, separated using SDS-PAGE (10%) and transferred to a nitrocellulose filter (0.2 μm, Bio-Rad, USA) by electrophoresis (Trans-Blot, Bio-Rad, USA) using the manufacturer’s instruction. Non-specific binding sites were blocked with 5% (w/v) skim milk powder in Tris buffered saline (145 mM NaCl, 20 mM Tris, pH 7.4) (SMTBS) overnight at 4 °C. All serum was used at a 1:5000 dilution in TBS containing 0.1% (v/v) Tween and 5% (w/v) skim milk powder. Affinity purified antibodies were used at a concentration of 1 μg/ml. Detection of bound antibody was achieved through the application of a 1:2000 dilution of polyclonal donkey anti-sheep IgG-HRP conjugate (Sigma, USA) diluted in PBST containing 5% (w/v) skim milk powder. The antibodies on the paper were detected and visualised by Enhanced Chemiluminescence (ECL; Amersham Biosciences).

### 2.10 Oncosphere killing

Adult *E. granulosus* were harvested as described by Heath and Lawrence [18]. Whole worms were briefly homogenised and passed through an 80 μm sieve to remove debris. Eggs were suspended and washed in PBS. Eggs were resuspended in 1% pepsin (Sigma, USA) and 1% (v/v) concentrated HCl in 0.85% saline. The suspension was gently mixed at 37 °C for 1 h. Following centrifugation at 500 g, the supernatant was discarded and the tube filled with a solution of 1% pancreatin (Sigma, USA), 1% NaHCO_3_ and 5% sheep bile. The suspension was gently mixed at 37 °C for 30 min.

The oncospheres were centrifuged (1000 × *g* × 2 min) and the supernatant discarded. Oncospheres were separated from the embryophoric blocks by thorough mixing in Percoll Cultivation Medium (100 ml 10× NCTC 135 (Sigma, USA), 10 ml 200 mM Glutamine, 100 mg gentamicin, 900 ml Percoll (Sigma, USA)) and centrifuging the mixture at 1000 × *g* for 10 min. The activated oncospheres were removed from the top of the solution and added to RPMI 1640 (Gibco, Invitrogen, USA), centrifuged and washed twice in RPMI culture medium. The number of activated oncospheres was estimated using an improved Neubauer haematocytometer counting chamber at 200 fold magnification.

Oncosphere cultures were propagated in 96-well flat bottomed Nunc Culture plates. Affinity purified antibodies were serially diluted from 68 μg/ml to 0.85 μg/ml. Each well contained 100 μl of affinity purified antibodies, 100 μl of RPMI culture medium, approximately 20 activated oncospheres and 50 μl of serum from a 6 month old naïve Merino wether. Penicillin, gentamicin and amphotericin were added to the cultures to give a final concentration of 50 U/ml, 50 μg/ml and 125 ng/ml respectively. All cultures were performed in duplicate and plates were cultured in a 5% CO_2_ atmosphere at 37 °C in a humidified incubator. Serially diluted sera from sheep immunised with EG95-GST and from naïve sheep were used as controls within each culture plate. After 10 days, the number of living oncospheres was counted and compared with the number surviving in the naïve serum samples. Results were expressed as the total number of oncospheres surviving within a culture well.

## 3 Results

**Fig. 1:**
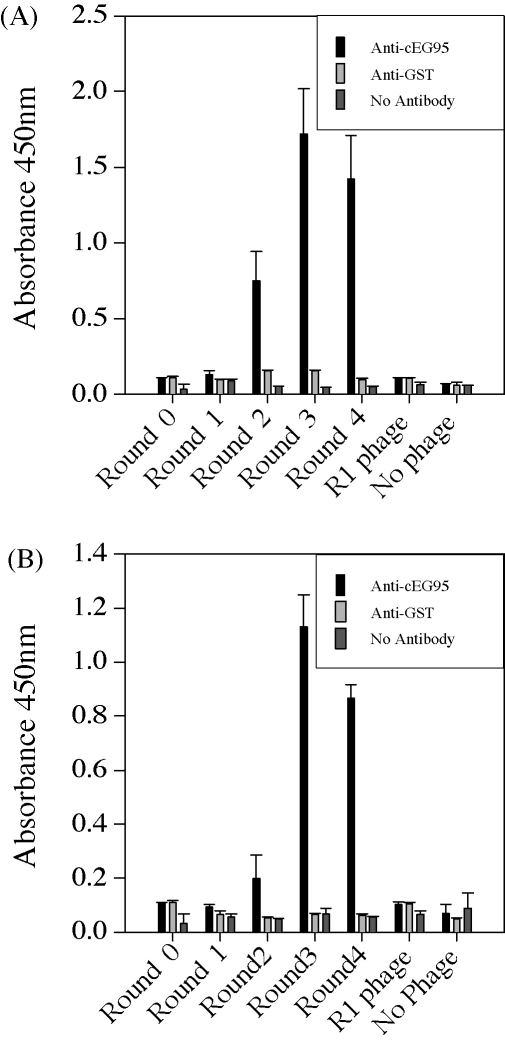
Indirect ELISA showing the enrichment of specific phage during the affinity selection process. Phages were affinity selected on anti-cEG95 using Library 1 (A) and Library 2 (B). Pooled phages were amplified and 10^10^ TU/ml were added to microtitre wells preadsorbed with anti-cEG95, anti-GST or skim milk powder. Phages were detected with anti-M13 HRP monoclonal antibody. R1 phage is a clone selected randomly from the library without any selection pressure.

Affinity purified polyclonal anti-cEG95 and anti-GST antibody were used to affinity select peptides from the 20-mer and 12-mer random peptide libraries. The progress of the affinity selection procedure was monitored by performing a phage indirect ELISA on an aliquot from each round of panning. The phage pools were examined for binding to anti-cEG95 antibody, anti-GST antibody and a zero antibody “mock” ELISA to observe evidence that phage were being selected for binding to the target antibody (Fig. 1).

Both experiments resulted in an increase in reactivity toward the target antibody over the first three rounds of panning. The reactivity toward the non-target antibody showed a slight increase during these panning rounds. Where no antibody was present in the ELISA, there was no increase. The fourth round of panning against anti-cEG95 resulted in a slight decrease in reactivity whereas the fourth round of panning against anti-GST resulted in a slight increase in reactivity (data not shown). The unselected library (Round 0) and R1 phage displayed similar levels of reactivity to the background (no phage) reactivity.

Twelve phage clones from the 20-mer library—E1, E2, E3, E6, E14, E20, E30, E33, E45, E55, E68 and E114, and eight from the 12-mer library—E91, E99, E100, E104, E105, E106, E108 and E110 were selected against anti-cEG95 and shown by DNA sequencing to contain different peptides (Table 1).

The amino acid sequences of the selected peptides were examined for homology to EG95. Phage clone E14 was found to display five residues that aligned with the EG95 sequence (Table 1). None of the other peptides could be aligned to the sequence of EG95.

The phage clone G1, which was selected with affinity purified anti-GST antibodies, demonstrated significant homology to the sequence of GST. Four residues over a seven residue sequence were the same in both G1 and GST.

One phage clone, R1, was chosen at random from the library without any affinity selection. There was no apparent sequence homology between this peptide and either EG95 or GST and this peptide was used as a negative control in subsequent experiments.

**Fig. 2:**
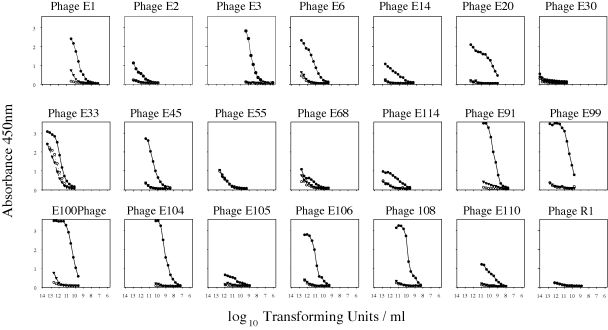
Binding of phage clones to anti-cEG95 (●), anti-GST (▾) and no antibody (○). The binding was quantified by the Phage Capture ELISA. Abscissa values are the common log of the phage concentration.

The relative avidities of phage toward anti-cEG95 and anti-GST antibodies were measured using phage indirect ELISA. The individual phage clones were titrated against anti-cEG95, anti-GST or with no antibody present (Fig. 2) enabling a determination as to which of the phage clones selected by panning against anti-cEG95 preferentially bound to anti-cEG95 antibody.

Selected phage clones were then subjected to a competitive phage indirect ELISA to determine if the binding to anti-cEG95 antibody could be inhibited by EG95-MBP. EG95-MBP was used as the inhibitor in these assays rather than the protein that the anti-sera was raised against (EG95-GST). This was to assess the EG95 component without having the results confounded by the possibility that the phage were binding to anti-GST antibodies.

**Fig. 3:**
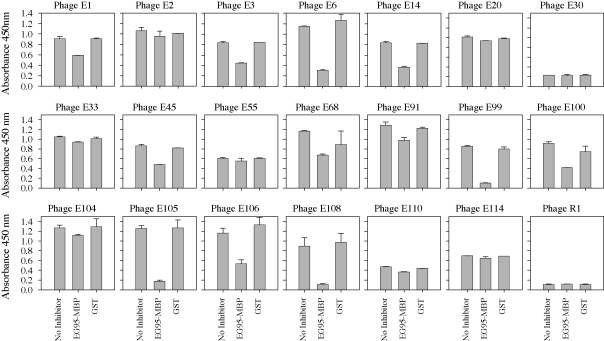
Competitive phage indirect ELISA. Anti-cEG95 antibodies were adsorbed onto microtitre plates and a mixture of phage and protein competitor (EG95-MBP or GST) at a final concentration of 250 μg/ml in PBS with 5% skim milk powder. Phage binding was measured. Displayed values are the mean ± SEM of duplicate wells.

The results indicate that phage clones E2, E20, E30, E33, E55, E110 and E114 were not inhibited from binding to anti-cEG95 antibodies by either EG95-MBP or GST (Fig. 3). The remaining phage clones were inhibited from binding to anti-cEG95 by EG95-MBP and could be divided into two groups (i) those that are inhibited from binding to a level that approached complete inhibition—E99, E105 and E108, and (ii) those that were partially inhibited—E1, E3, E6, E14, E45, E68, E91, E100, E104 and E106.

**Fig. 4:**
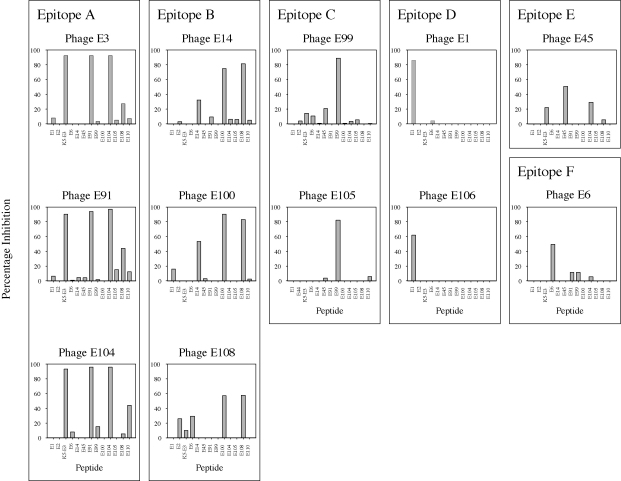
Effect of synthetic peptides on the binding of phage clones to anti-cEG95 antibody. Individual panels each represent a single phage clone. The abscissas correspond to thirteen synthetic peptides. The ordinates are the percentage of inhibition that individual peptides cause relative to the addition of no inhibitor. Putative Epitopes A–F represent phages displaying similar patterns of inhibition by synthetic free peptides.

Thirteen synthetic peptides were examined for their ability to compete with twelve phage clones for binding to anti-cEG95 antibodies. This experiment was to determine whether there were groups of peptides that mimicked the same epitope(s) on EG95. The results (Fig. 4) of these competitive phage indirect ELISAs indicate that with the exception of E105, each peptide competed with its corresponding phage clone for binding to the anti-cEG95 antibodies. Peptide E106 was not synthesised. Based on the pattern of peptide competition we placed the phage clones we examined into groups of putative epitopes:

- Epitope A, clones E3, E91 and E104 were each inhibited from binding to anti-cEG95 antibodies by peptides K_5_-E3, E91 and E104.
- Epitope B, clones E14, E100 and E108 were each inhibited from binding to anti-cEG95 antibodies by peptides E100 and E108. Peptide E14 showed a diminished degree of inhibition to these phage clones.
- Epitope C, clones E99 and E105 were each inhibited from binding to anti-cEG95 antibodies by peptide E99. Peptide E105 did not inhibit its corresponding phage clone, nor did it inhibit phage clone E99.
- Epitope D, clones E1 and E106 were each inhibited from binding to anti-cEG95 antibodies by peptide E1.

Phage E45 was inhibited from binding to anti-cEG95 antibodies by peptide E45, and to a diminished extent by peptides E104 and K_5_-E3. Phage E6 was inhibited from binding to anti-cEG95 antibodies by peptide E6. Consensus motifs were found in the amino acid sequences present in epitopes A, B, C and D (Table 2).

**Fig. 5:**
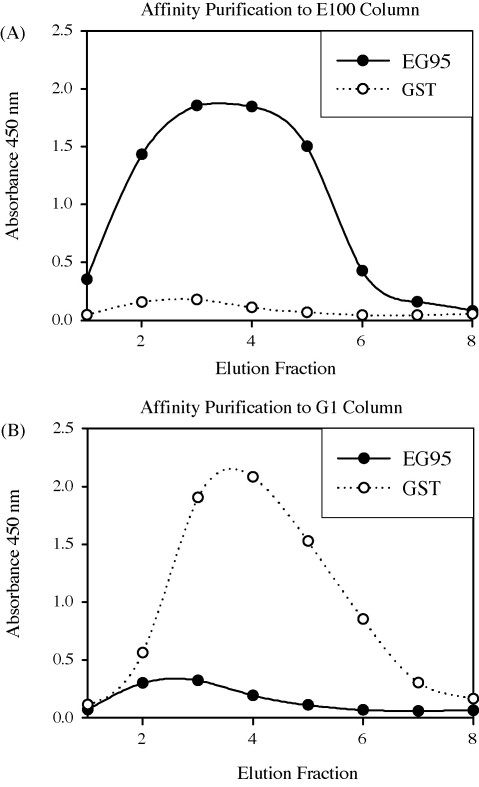
Antibody capture ELISA results of affinity purification fractions. Affinity purified antibody fractions were examined for ability to bind EG95-MBP (●) or GST (○). The antibodies affinity purified to E100 peptide are represented in panel A. The antibodies affinity purified to G1 peptide are represented in panel B.

Peptide E100 was the most effective of the selected peptides for use as an affinity purification ligand (data not shown) and together with peptide G1 was chosen as an affinity purification ligand. NHS-Sepharose beads were linked to peptide E100 and peptide G1 to produce two affinity purification columns. Affinity purification of antibodies from sheep immunised with EG95-GST was carried out using these two columns as targets. Eluted antibodies were neutralised and collected as 500 μl fractions. The reactivity of each eluted fraction to EG95-MBP and GST in antibody indirect ELISAs are shown in Fig. 5. The fractions that were eluted from the E100 peptide column showed specific reactivity to EG95-MBP with a peak in fractions 3 and 4; there was low reactivity to GST. The fractions eluted from the G1 peptide column showed specific reactivity to GST, with a peak of reactivity in fraction 4, and low reactivity to EG95-MBP.

**Fig. 6:**
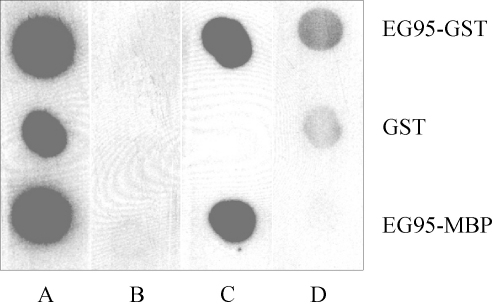
Dot blot assay. Recombinant proteins EG95-GST, GST and EG95-MBP were blotted on to nitrocellulose membrane. The proteins were probed with pooled anti-sera from sheep immunised with EG95-GST (column A), naïve pooled sera (column B), antibodies affinity purified on E100-NHS-Sepharose (column C) and antibodies affinity purified on G1-NHS-Sepharose (column D).

Dot blot assays were used to further assess the specificity of the antibodies affinity purified by each of the two peptides, E100 and G1, and also anti-EG95-GST anti-sera from which the antibodies were originally purified by affinity chromatography. Pooled sera obtained from sheep prior to immunisation were also examined by dot blot assays. The results (Fig. 6) show that pooled anti-sera obtained from sheep immunised with EG95-GST reacted with EG95-GST, GST and with EG95-MBP. Antibodies affinity purified on peptide E100 reacted exclusively with proteins that contained EG95, that is, EG95-GST and EG95-MBP, and antibodies affinity purified on peptide G1 reacted with EG95-GST and GST. Pre-immune pooled sera showed only background levels of reactivity in this assay.

**Fig. 7:**
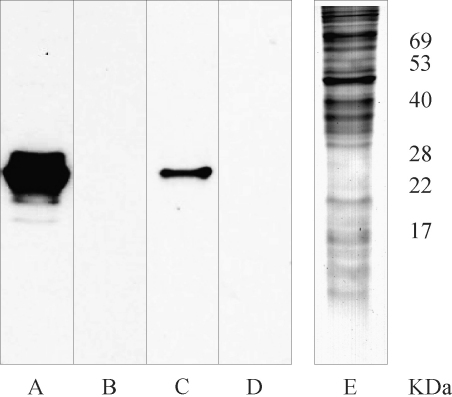
Western blots of *E. granulosus* oncosphere antigens reacted with sheep anti-sera to EG95-GST (lane A), naïve sera (lane B), antibodies affinity purified on E100-NHS-Sepharose (lane C) and antibodies affinity purified on G1-NHS-Sepharose (lane D). Lane E is a Coomassie stained SDS-PAGE of the oncosphere antigens.

The antibodies that were affinity purified on peptide E100-Sepharose or G1-Sepharose were then assessed for the ability to react with native antigens in *E. granulosus* using a Western blot assay. The pooled anti-EG95-GST anti-sera reacted strongly with an oncosphere antigen with a relative molecular weight of approximately 23 kDa (Fig. 7). The antibodies affinity purified on peptide E100 also reacted with this same antigen whereas antibodies affinity purified on peptide G1 or pre-immune anti-sera did not react with any oncosphere antigens.

**Fig. 8:**
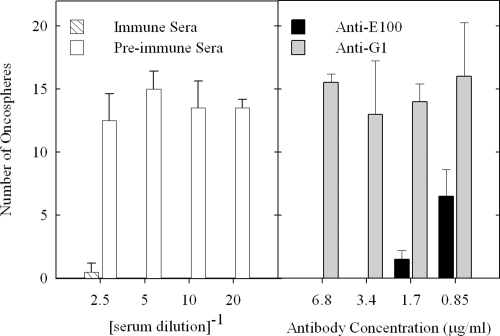
Survival of *E. granulosus* oncospheres *in vitro* in the presence of pooled anti-EG95-GST anti-sera (hatched bars), pre-immune sera (white bars), antibodies affinity purified on peptide E100 (black bars) and peptide G1 (grey bars). Sera in the test wells were diluted from 1:2.5 through to 1:20. Affinity purified antibodies were diluted from 6.8 μg/ml to 0.85 μg/ml. Assays for each dilution were performed in duplicate. Each individual bar represents the number of oncospheres (mean ± SEM) surviving after 10 days of culture.

Anti-EG95-GST, anti-E100 antibody and anti-G1 antibody preparations were assessed for their ability to kill *E. granulosus* oncospheres *in vitro*. Pooled sera obtained from sheep immunised with EG95-GST and corresponding pre-immune sera were used as controls. The results are shown in Fig. 8. The anti-EG95-GST anti-sera were observed to kill oncospheres, with only a single oncosphere surviving. Oncosphere killing was observed in all cultures containing antibodies purified on E100 peptide. The killing was complete for antibody concentrations greater than or equal to 3.4 μg/ml. Antibodies affinity purified on G1 peptide were unable to kill oncospheres as was pre-immune sera.

## 4 Discussion

The data presented here indicates that peptide E100 mimics of a conformation-dependant and protective epitope of EG95. The peptide could be used to purify a sub-population of antibodies produced following immunisation with EG95-GST and these affinity purified antibodies could bind to native EG95 and kill *E. granulosus* oncospheres. The results demonstrate that random peptide phage display libraries can be used to identify molecular mimics of EG95 and, in this case, putatatively protective epitopes.

The random peptide phage display libraries that we used contained between 10^8^ and 10^10^ unique peptides and were used to select peptides that bound anti-cEG95 antibodies. The selection process does not distinguish between those peptides that bind to the complementarity-determining regions and those that bind to other parts of the antibody. The method of biopanning that we used was able to select peptides from both phage display libraries and enrich the specific binding phage during each of the first three rounds of panning.

The slight decrease in the ELISA signal following the fourth round of biopanning may have been due to the presence of a greater abundance of phages with higher avidity for the target in the third round pool when compared to the fourth round pool. Some phage displayed peptides can confer a selective growth advantage (or disadvantage) for the bacterial host [19]; possibly therefore phage obtained from the fourth round of biopanning may have had such a growth advantage but lower avidity toward the selecting antibody. The relatively small decrease in ELISA signal would suggest that this change in the ratio of selected phages in the pool was not significant, and would be unlikely to have eliminated any phage clones that were important in providing the ELISA signal. Other possible explanations for the decrease in ELISA signal in the final round of panning include the possibility of inaccuracies in the phage titre or increased protease activity in this sample. These possibilities again do not diminish the fact that specific affinity purification occurred in this final round of biopanning.

Only one of the selected displayed peptides showed sequence homology to EG95. This area of EG95 was not one of the areas thought to contain linear epitopes [10]. None of the sequences of the remaining selected peptides aligned with the amino acid sequence of EG95. *Prima facie* this may appear to indicate that the use of polyclonal antibodies with linear epitope specificities removed was successful at overcoming the bias toward selecting peptides that bind to linear epitopes.

The indirect ELISA used here allowed phage to be differentiated into those that bound specifically to anti-EG95 antibodies and those that did not. A number of the phage clones appeared to bind specifically to anti-EG95 antibodies (when compared to anti-GST antibodies), but did not have absorbances as high as others in the ELISA. Presumably these phage displayed peptides were reacting with a sub-population of antibody that was of minor specificity within the anti-EG95 antibody population. Competitive phage indirect ELISA using EG95-MBP allowed some phage clones to be eliminated as potential mimetics (Fig. 3). These phage clones could not be inhibited from binding to anti-cEG95 by the addition of EG95-MBP. There are two possible causes for these peptides to be unable to be competitively inhibited by EG95. The first possibility is that the phage displayed peptide does not bind to the same paratope as EG95, such peptides are not (by definition) mimotopes of EG95. A second possibility is that these peptides have a significantly higher avidity for the antibody than does EG95. When these phage clones were titrated in the phage indirect ELISA, however, they had some of the lowest reactivities of the selected phages. In order for these phage clones to have a high affinity for anti-cEG95 the peptides are likely to be binding to an antibody sub-population of very minor specificity. By eliminating those peptides that were not specific for anti-cEG95 and those that could not be inhibited from binding to anti-cEG95 by EG95-MBP there are twelve peptides that act as conformation-dependant epitope mimics.

A portion of the anti-EG95 antibodies raised by immunising sheep with EG95-GST was able to be purified using peptide E100 linked to NHS-Sepharose. Similar experiments using E45, E91, E99, E104 and E108 were unable to extract any detectable antibodies (data not shown). This highlights one of the important aspects associated with the use of peptide mimetics, namely that the conformation that the peptide assumes may be altered depending on the environment in which the peptide is placed. Peptide E100 was the only peptide examined that was capable of being used to extract anti-EG95 specific antibodies. These antibodies raised against EG95 were also shown to be lethal to *E. granulosus* oncospheres *in vitro*. Inhibition studies using these antibodies showed that they were capable of being inhibited from binding to EG95-MBP by the presence of free synthetic peptide E100. The antibodies that were affinity purified were capable of binding specifically to EG95 both in the native and recombinant forms. Since the affinity purified antibodies are lethal to oncospheres *in vitro*, E100 does mimic a protective epitope of EG95. Peptides E108 and E14 both interact with the same antibodies as E100. E14 is capable of inhibiting affinity purified anti-E100 antibodies from binding to EG95 (data not shown). Further information about this particular epitope may be deduced by comparing the amino acid sequences of these three peptides. The sequence KXNNDPXAA is a likely consensus sequence for this particular epitope. It would be of further interest to produce a series of synthetic peptides containing this motif along with alanine/glycine substitutions of the common residues in order to confirm which residues are essential for this peptide to act as an epitope mimic.

Further research will need to be performed if a peptide vaccine is to be discovered that can protect sheep from infection with *E. granulosus*. The potential for development of a peptide-based vaccine is encouraged by the success that has been achieved with the development of a phage-based vaccine against cysticercosis [20]. The data presented in this paper points toward at least six antibody specificities being present in antibody purified to conformation-dependant epitopes of EG95, presumably relating to six conformation-dependant epitopes on EG95. One of the selected peptides is capable of affinity purifying antibodies that are lethal to *E. granulosus in vitro*. This peptide has the potential to be used as the basis for determining whether protective antibodies have been produced in sheep vaccinated with EG95.

## Figures and Tables

**Table 1:** Deduced amino acid sequence of the displayed peptides of phage clones selected after four rounds of biopanning with anti-cEG95 antibody as a target. Alignment of peptide E14 to EG95. Aligned residues indicated in bold.

E1	CIFPLCLGRPIAASYPPKTR
E2	LNSRHTDKGTVQFALIAVRG
E3	YVALQGSMFDRVRVFWMARG
E6	ACSDRFLRRPMCLPPHVVFL
E14	GFGKDITTGHSFTHKSNNDP
E20	TTGNLSLDLLIATRFSSHGK
E30	IHTDRPAWKYVGALPVIHVR
E33	NSNFTVHSNYWYWNRYIPPV
E45	TNLLYMLNTEAQYKALFMRK
E55	ILPNPIHHTWLPFKLSHNLP
E68	GCSYPMCRFNVVRFTGLSLL
E114	GLFDLTNYYYLSRGTQIKGT
E91	NRLFDRVDRFYA
E99	GKPGWLLDNVAL
E100	HYKWLNDPLAAW
E104	TLFGRMEHYFN
E105	ISTSKPAWKLAN
E106	CIPPLCKLRLHE
E108	LDKRNNDPFHLP
E110	SVSVGMKPSPRP

E14	**GF**GK**DI**T**T**GHSFTHKSNNDP
EG95	_110_KKTIL**GF**TV**DI**E**T**PRAGKKESTVMTSGSAL_139_

**Table 2:** Alignment of amino acid sequences within each group determined in the peptide competition ELISA. Consensus sequences were determined to be present when two or more amino acid residues aligned. Epitope labels correspond to groupings found in Fig. 4. Underlined residues derived from phage pIII protein.

Epitope group	Phage	Amino acid sequence
Epitope A	E3	YVALQGSM**FDRV**RV**F**WMARG
	E91	NR**LFDRV**DR**F**YA
	E104	T**LF**G**R**MEHYFN
	**Consensus A**	**LFDRV**XX**F**

Epitope B	E14	GFGKDITTGHSFTH**K**S**NNDP**G**AA**G
	E100	HY**K**WL**NDP**L**AA**W
	E108	LD**K**R**NNDP**FHLP
	**Consensus B**	**K**X**NNDP**X**AA**

Epitope C	E99	G**KP**G**W**L**L**D**N**VAL
	E105	ISTS**KP**A**W**K**L**A**N**
	**Consensus C**	**KP**X**W**X**L**X**N**

Epitope D	E1	**CI**F**PLC**LG**R**PIAASYPPKTR
	E106	**CI**P**PLC**KL**R**LHE
	**Consensus D**	**CI**X**PLC**XX**R**
